# Parkinson's Symptoms and Caregiver Burden and Mental Health: A Cross-Cultural Mediational Model

**DOI:** 10.1155/2019/1396572

**Published:** 2019-12-01

**Authors:** Erin R. Smith, Paul B. Perrin, Carmen M. Tyler, Sarah K. Lageman, Teresita Villaseñor

**Affiliations:** ^1^Department of Psychology, Virginia Commonwealth University, USA; ^2^Department of Physical Medicine and Rehabilitation, Virginia Commonwealth University, USA; ^3^Department of Neurology, Parkinson's and Movement Disorders Center, Virginia Commonwealth University, USA; ^4^Hospital Civil Fray Antonio Alcalde, Mexico; ^5^Department of Neurosciences, University of Guadalajara, Mexico

## Abstract

Informal caregivers are critical in the care of individuals with Parkinson's disease (PD) and spend substantial time providing care, which may be associated with negative caregiver outcomes such as burden and mental health issues. Although research in the United States and Europe has generally supported these relations, there is very limited research on PD caregiving in Latin America. The current study examined the following connections in a sample of PD caregivers from the United States (*N* = 105) and Mexico (*N* = 148): (a) PD-related impairments (motor and nonmotor symptoms) and caregiver burden, (b) caregiver burden and caregiver mental health, and (c) PD-related impairments and mental health through caregiver burden. Study results uncovered significant relations among PD-related impairments, caregiver burden, and caregiver mental health. Further, caregiver burden fully mediated the relation between PD-related impairments and caregiver mental health at both study sites. Findings highlight a number of important intervention targets for caregivers and families, including caregiver burden and mental health.

## 1. Introduction

Parkinson's disease (PD) is a progressive neurodegenerative condition that leads to physical disability [1] and cognitive impairment [2] over time, both of which may limit an individual's independent functioning. PD is the second most common progressive neurodegenerative disease in the United States [3], affecting approximately 1% of individuals over the age of 60 [4]. Recent estimates suggest that by 2030, there will be approximately 1.2 million individuals in the United States living with PD [5]. Similarly, due to its aging population [6], rates of PD are likely to increase rapidly in Latin America, with estimates suggesting the prevalence of PD will double in Mexico within 20 years [7] since rates of PD increase with age [8]. There are an estimated 129,124 individuals living with PD in Central Latin America, 30,717 in Andean Latin America, and 131,748 in Tropical Latin America [9]. However, despite the high prevalence and increasing rates of PD in Latin America, very little is known about either PD patient or caregiver experiences in the region and how experiences may differ from those in the United States.

PD is characterized by its classic motor symptoms, including akinesia (loss or impaired voluntary movement), bradykinesia (slowness of movement), resting tremor (shaking while in a relaxed state), and postural instability [10]. Individuals may present with additional motor symptoms, including gait problems [11] as well as reduced facial expression [12]. By the time an individual is 10 years postdiagnosis, the individual will present with a number of nonmotor symptoms [13] that can include depression, cognitive impairment, apathy, anxiety, sleep disruption, dementia, and psychosis [14].

Given the progressive nature of the disease, impairment increases over time, leading most individuals living with PD to require the assistance of a caregiver. This care is often provided by an informal caregiver, an individual who does not receive financial compensation for caregiving and is usually a family member [15, 16]. Informal caregivers support the individual with PD by performing a number of physical, social, and emotional tasks, which may include assisting with personal care and activities of daily living (e.g., bathing, feeding, and administering medications), transportation and mobility assistance (e.g., getting in and out of bed), providing social and emotional support, and financially supporting the individual [17–19]. Providing care may come at a significant cost to the caregiver, such as giving up a career, leisure activities, or social activities to take care of the individual with PD [17], and negative consequences for the caregiver may include having a lower quality of life than the general population [20, 21]. These factors contribute to the caregiver becoming what has been described as an “invisible patient,” and the toll of caregiving on a caregiver's life may diminish the overall effectiveness of the care they provide [14].

These substantial life changes may result in caregiver burden, a multidimensional construct that has been operationalized in a number of ways. For example, Zarit et al. [22] describe caregiver burden as encompassing the adverse effects caregiving may have on an individual's emotional, financial, social, physical, and spiritual function which may engender feelings of discomfort due to the demands, time constraints, duties, and difficulties of providing care. Successive researchers have built upon this definition by adding the internal conflict that caregivers may experience when they are unable to fulfill their personal needs due to caregiving [23], as well as including the reactions informal caregivers may have to the emotional, social, physical, and financial difficulties that result from providing care [24]. Caregiver burden is critical to examine, as it may lead to a reduced sense of well-being and burnout [25].

One of the first studies to examine motor symptoms and caregiver burden was conducted by Carter et al. [26], who found that individuals who were rated by clinicians to be in the later Hoehn and Yahr [27] stages of PD had spousal caregivers with higher caregiver strain than those in early stages. Other studies generally support the relationship between motor impairments and burden. For example, Martínez-Martín et al. [21] examined Hoehn and Yahr [27] staging and caregiver burden in a sample of Spanish caregivers and found that PD severity was a primary predictor of caregiver burden. Increasing attention has also been given to the nonmotor symptoms of PD [14], which may be difficult for caregivers to address. Anywhere from 25 to 40% of individuals living with PD eventually develop dementia [28] and may experience related deficits in attention, memory, and executive functioning [14]. Leroi et al. [29] found that caregiver burden was significantly higher in caregivers who cared for an individual with PD who had dementia than those without cognitive impairment or with only mild cognitive impairment, suggesting that dementia is independently associated with increased caregiver burden.

The mental health of PD caregivers has been found to be lower than that of the general population [30], suggesting that caregiving may take a toll on an individual's mental health. Depression and anxiety specifically may be critical to examine as they can elicit cognitive biases that engender greater feelings of caregiver burden [14, 31]. At least one study has suggested that the link between depression and caregiver burden may be particularly critical in PD compared to other neurological diseases (e.g., Alzheimer's disease [32]).

To date, very little research has examined PD in regions outside of North America and Europe. The majority of research conducted outside of these regions has focused on data derived from medical records or drug consumption data [33]. This is problematic for low-to-middle-income countries, as these estimates inherently exclude individuals who are unable to obtain medical care or prescription drugs to treat PD [34]. Further, these studies have also not considered the unique culturally determined treatment practices and varying access to care for PD throughout the world [35]. As such, there is a critical need to examine PD caregiving in diverse regions of the world, such as Latin America. Given the rapidly aging population in both the United States [5] and Mexico [7], rates of PD are likely to rise in both countries, suggesting that the number of individuals providing informal care will also increase. As a result, the current study examined the following connections in two samples of PD caregivers from the United States and Mexico: (a) PD-related impairments (motor and nonmotor symptoms) and caregiver burden, (b) caregiver burden and caregiver mental health, and (c) PD-related impairments and mental health through caregiver burden. The current study presents the results of a secondary analysis of data from another manuscript comparing PD caregiver and patient characteristics in the United States and Mexico [36]. This other manuscript found that caregivers in the United States site were older than those in Mexico, spent fewer hours per week providing care, provided care to an older individual with PD, and had provided care for a longer duration of time, although there were no differences in the number of individuals who assisted the caregiver in providing care. In the current study, it was hypothesized that caregiver burden would mediate the association between PD-related impairments and mental health across both sites.

## 2. Method

### 2.1. Participants

This study used cross-sectional data that were collected from PD caregivers from the Hospital Civil Fray Antonio Alcalde of the University of Guadalajara in Guadalajara, Mexico, and the Parkinson's and Movement Disorders Center (PMDC) at Virginia Commonwealth University in Henrico, Virginia. Both centers offer interdisciplinary models of health care for patients as well as services for caregivers. The Hospital Civil Fray Antonio Alcalde also offers education programs and emotional support groups led by psychologists and lodging and food support for caregivers. To be eligible for this study, participants had to have met the following inclusion criteria: (a) identify as a caregiver of an individual diagnosed with PD, (b) be at least 18 years of age, and (c) be fluent in either English (for the United States site) or Spanish (for the Mexico site). Please see Table 1 for PD caregiver and patient characteristics.

In the United States sample (*N* = 105), the majority of caregivers self-identified as women (68.6%). Caregivers had a mean age of 68.73 (SD = 8.36) and were predominantly spouses or partners (93.3%) of individuals with PD. On average, caregivers had provided care for 49.05 months and 60.43 hours per week. The majority of individuals self-identified as White/European American (92.4%), followed by Asian/Asian American/Pacific Islander (2.9%), Black/African American (non-Latino; 2.9%), multiracial/multiethnic (1.0%), or other identity (1.0%). Of the sample, 25.7% had a high school education or equivalent, 2-year technical degree (11.4%), 4-year college degree (33.3%), master's degree (21.9%), or doctorate degree (7.6%).

In the Mexico sample (*N* = 148), the majority of caregivers self-identified as women (76.4%) and had a mean age of 53.66 (SD = 14.96). Over half of the caregivers were spouses or partners of the individual living with PD (51.4%). On average, caregivers provided care for 52.38 (SD = 49.22) months and 107.39 (SD = 61.34) hours per week. Of the sample, 4.7% had no formal education, 58.1% had an elementary school education, 5.4% had a high school education or equivalent, 13.5% had a 2-year technical degree, 16.2% had a 4-year college degree, and 2.0% had a master's degree.

### 2.2. Measures

#### 2.2.1. PD Impairments

PD-related impairments were assessed using the Movement Disorder Society-Unified Parkinson's Disease Rating Scale (MDS-UPDRS; [37]). Participants were instructed to answer this questionnaire based on their observations and experiences with the individual living with PD. Participants responded to two subscales within the questionnaire: Part I (nonmotor aspects of experiences of daily living) and Part II (motor aspects of experiences of daily living). Participants respond to each item on a Likert-type scale that ranges from 0 (normal: no problems present) to 4 (severe: problems are present and preclude the patient's ability to carry out normal activities or social interactions or to maintain previous standards in personal or family life). In the present study, total scores for each subscale were created by summing scores for each item within the subscale. The nonmotor experiences of the daily living subscale has acceptable internal consistency (*α* = .79), and the motor experiences of daily living has good internal consistency (*α* = .90; [37]). Additionally, for symptom-based exploratory analyses, scores were calculated for the following subdomains on the MDS-UPDRS: cognitive/mental health (items 1.1-1.6), sleep and fatigue (items 1.7, 1.8, and 1.13), bowel and bladder (items 1.10 and 1.11), pain (item 1.9), lightheadedness (item 1.12), activities of daily living (items 2.5-2.8), physical impairment (items 2.9-2.13 and 1.12), and speech/oral (items 2.1-2.4). The Movement Disorder Society (MDS) has also translated and validated the scale in Spanish, with the nonmotor experiences of daily living demonstrating acceptable internal consistency (*α* = .79) and the motor experiences of daily living demonstrating good internal consistency (*α* = .92; [38]). In the current study, the nonmotor subscale demonstrated good internal consistency in the Mexico (*α* = .80) and the United States sample (*α* = .85). The motor subscale also demonstrated good internal consistency in the Mexico (*α* = .88) and the United States sample (*α* = .90). No specific instructions were given regarding responding when the patient was “on” or “off” medication status.

#### 2.2.2. Caregiver Burden

The short version of the Zarit Burden Inventory [39] was used to assess caregiver burden. Participants responded to the 12-item version of the ZBI on a Likert-type scale from 0 (never) to 4 (nearly always), where higher scores indicate higher levels of caregiver burden. The full version of the ZBI has previously been validated in caregivers of individuals living with Parkinson's disease [21]. The ZBI has also been validated in Spanish-speaking individuals and demonstrated good internal consistency (*α* = .92; [40]). In the current study, the measure demonstrated good internal consistency in the Mexico (*α* = .86) and United States samples (*α* = .91).

#### 2.2.3. Mental Health

To compute the mental health variable, *z*-scores from the Patient Health Questionnaire-9 (PHQ-9; [41]), assessing for depressive symptomatology, and the Generalized Anxiety Disorder-7 (GAD-7; [42]), assessing for anxiety, were averaged.

*(1) Depression*. Participants responded to the 9-item PHQ-9 [41] on a Likert-type scale from 0 (not at all) to 3 (nearly every day), where higher scores correspond to greater depressive symptomatology. The PHQ-9 has previously been validated in Spanish-speaking individuals and demonstrated good internal consistency (*α* = .92; [43–45]). In the current study, the PHQ-9 had good internal consistency in the Mexico (*α* = .81) and United States samples (*α* = .82).

*(2) Anxiety*. Participants responded to the 7-item GAD-7 [42] on a Likert-type scale from 0 (not at all) to 3 (nearly every day). Participants' scores may range from 0 to 21, with higher scores indicating greater levels of anxiety. The GAD-7 has previously been translated and validated in Spanish and demonstrated excellent internal consistency (*α* = .92; [46]). In the current study, the GAD-7 demonstrated good internal consistency in the Mexico (*α* = .88) and United States samples (*α* = .90).

### 2.3. Procedure

The protocol for the current study was reviewed and approved by the Institutional Review Boards at both data collection sites, Virginia Commonwealth University and the Hospital Civil Fray Antonio Alcalde. Participants were recruited by research assistants at both sites using written and verbal advertisements, predominantly from waiting rooms but also via clinician referral after medical appointments. Email advertisements were also sent to a listserv at the PMDC at Virginia Commonwealth University. At each data collection location, interested individuals were provided with information on the study in the respective clinic and provided informed consent prior to enrolling in the study. Participants were screened for eligibility, and if eligible, completed all study measures. The protocol was orally administered at the Hospital Civil Fray Antonio Alcalde site to collect demographic and questionnaire data in order to account for higher rates of illiteracy than at the United States site. The oral interview took approximately an hour. Participants from the PMDC completed all survey measures independently using pencil and paper. Completion of study measures took participants approximately the same amount of time.

### 2.4. Data Analyses

Four mediational models were created to determine if caregiver burden mediates the relationship between PD-related impairments and caregiver mental health. The first model used motor impairments as a predictor and a second model used nonmotor impairments as a predictor. Each of these models was run separately by site for a total of four mediational models using the PROCESS macro [47]. This macro utilizes the Preacher and Hayes [48, 49] asymptotic bootstrapping approach. In this approach, a large number of samples are taken from the data, and by sampling with replacement, the indirect effect from each sample is calculated. A sample of 5,000 bootstrap samples was taken as recommended by Preacher and Hayes [48, 49]. The indirect effect estimate was calculated by taking the mean of all of the indirect effects across the bootstrap samples. In this approach, statistical significance is determined by creating a confidence interval surrounding the indirect effect. The analysis used a 95% confidence interval with an *α* level of .05. The null hypothesis is rejected (i.e., no indirect effect) if the obtained confidence interval does not contain zero. All predictor variables (motor and nonmotor impairments, caregiver burden) were mean-centered prior to running analyses. Finally, an exploratory step-wise multiple regression was run predicting caregiver burden, wherein the first step included important demographic variables as predictors and the second step included symptom clusters of PD (cognitive/mental health, sleep and fatigue, bowel and bladder, pain, lightheadedness, activities of daily living, physical impairment, and speech/oral).

## 3. Results

### 3.1. Motor Impairments

Please see Table 2 for a summary of PD motor impairments. For the Mexico site, the overall model was significant, *F*(2, 145) = 5.54, *p* < .001, and *R*^2^ = .07. The direct path from motor symptoms to caregiver burden was statistically significant (*b* = .32, *p* < .001), as was the path from caregiver burden to caregiver mental health (*b* = .06, *p* = .0016). The motor impairment model demonstrated a mean bootstrap estimate of the indirect effect of *b* = .02. The obtained confidence interval did not contain zero (.01, .04), suggesting that caregiver burden mediates the association between motor impairments and caregiver mental health among caregivers from the Mexico site. Overall, this suggests that greater motor impairments predicted greater caregiver burden, which predicted greater mental health deficits among caregivers. The direct effect between motor impairments and mental health was *b* = −.01 and was not significant (*p* = .677), suggesting that caregiver burden fully mediated the association between motor symptoms and caregiver mental health.

Similar results were obtained for the United States site. The overall model was significant, *F*(2, 102) = 26.48, *p* < .001, and *R*^2^ = .34. The direct path from motor symptoms to caregiver burden was statistically significant (*b* = .44, *p* < .001), as was the path from caregiver burden to caregiver mental health (*b* = .14, *p* < .001). The motor impairment model demonstrated a mean bootstrap estimate of the indirect effect of *b* = .06. The obtained confidence interval did not contain zero (.04, .09), suggesting that caregiver burden mediated the association between motor impairments and caregiver mental health among caregivers from the United States site. Given that the direct effect between motor impairments and mental health was *b* = −.03 and was not significant (*p* = .097), this suggests that caregiver burden fully mediated the association between motor impairments and caregiver mental health.

### 3.2. Nonmotor Impairments

Please see Table 2 for a summary of PD nonmotor impairments. For the Mexico site, the overall model was significant, *F*(2, 145) = 6.46, *p* = .002, and *R*^2^ = .08. The direct path from nonmotor symptoms to caregiver burden was statistically significant (*b* = .48, *p* < .001) and the direct path from caregiver burden to caregiver mental health was significant (*b* = .05, *p* = .026). The nonmotor impairment model demonstrated a mean bootstrap estimate of the indirect effect of *b* = .02. The obtained confidence interval did not contain zero (.0036, .04), suggesting that caregiver burden mediates the association between nonmotor impairments and caregiver mental health among caregivers from the Mexico site. Similar to the prior model, these results suggest that greater nonmotor impairments predicted greater caregiver burden, which predicted greater mental health deficits among caregivers. The direct effect between nonmotor symptoms and caregiver mental health was *b* = .03 and was not significant (*p* = .172), suggesting that caregiver burden fully mediated the association between nonmotor impairments and caregiver mental health among caregivers from the Mexico site.

Again, similar results were obtained for the United States site. The overall model was significant, *F*(2, 102) = 25.63, *p* < .001, and *R*^2^ = .33. The direct path from nonmotor symptoms to caregiver burden was significant (*b* = .65, *p* < .001) and the direct path from caregiver burden to caregiver mental health was significant (*b* = .14, *p* < .001). The model demonstrated a mean bootstrap estimate of the indirect effect of *b* = .09. The obtained confidence interval did not contain zero (.05, .14), suggesting that caregiver burden mediated the association between nonmotor impairments and caregiver mental health among caregivers from the United States site. The direct effect between nonmotor impairments and mental health was *b* = −.03 and was not significant (*p* = .203), suggesting that caregiver burden fully mediated the association between nonmotor impairments and caregiver mental health.

### 3.3. Exploratory Analysis

In the step-wise regression predicting burden, the first step with demographic predictors (caregiver age, caregiver gender, hours per week providing care, months providing care, caregiver education, and number of individuals who assisted the caregiver in providing care) was statistically significant, *F*(6, 240) = 2.65, *p* = .017, and *R*^2^ = .06. Within the overall model, the only unique predictor was caregiver education (*β* = .17, *p* = .014). In the second step, PD symptom clusters were added as predictors, resulting in an overall statistically significant model, *F*(8, 232) = 14.70, *p* < .001, and *R*^2^ = .38. Within the overall model, the only unique predictor was caregiver cognitive/mental health symptoms (*β* = .35, *p* < .001).

## 4. Discussion

The current study examined PD caregivers in Henrico, Virginia, and Guadalajara, Mexico, and the relations among PD-related impairments, caregiver burden, and caregiver mental health. Results from both the motor and nonmotor impairments models suggest that caregiver burden fully mediates the association between PD-related impairments and caregiver mental health in both caregivers from the United States and from Mexico. Generally, prior research has supported the links among PD-related impairments and caregiver burden [14], caregiver burden and mental health [31], and PD-related impairments and mental health [20, 50]. To date, this is the first evidence that caregiver burden fully mediates the relations between both PD-related motor and nonmotor impairments and mental health. One possible explanation for these results is that as PD-related impairments become more severe, levels of caregiver burden increase, which in turn may lead to poorer mental health.

PD-related impairments, particularly nonmotor symptoms, were associated with deleterious outcomes for caregivers in the current study. Further, the exploratory multiple regression suggested that cognitive/mental health symptoms had the biggest impact on caregiver burden. These findings are similar to those of Schrag et al. [51], who found that PD caregiver burden was particularly related to nonmotor symptoms including depression, hallucinations, and confusion. The current findings in light of the previous research suggest that it may be important for health care providers of caregivers to be mindful of nonmotor PD-related impairments and how they may affect the psychosocial functioning and well-being of the caregiver. Interventions that target PD-related impairments may also be useful in reducing caregiver burden and have already received some support in the literature. For example, interventions that promote independence and functioning among individuals with PD may be associated with reduced caregiver burden. Recently, exercise interventions for individuals with PD have received attention. Oguh et al. [52] found that individuals with PD who exercised more than 150 minutes a week had better quality of life, physical function, and reduced disease progression, as well as less caregiver burden among their caregivers than those who were not regular exercisers.

Targeting nonmotor symptoms may also serve to reduce deleterious outcomes for caregivers, such as caregiver burden and mental health issues. For example, previous studies have shown that addressing dementia with cognitive enhancers has been associated with reduced caregiver burden [53, 54]. Prior research has also demonstrated that tailored cognitive behavioral therapy for individuals with PD with anxiety is associated with reduced caregiver burden [55]. Overall, these studies suggest that there is benefit in considering PD-related impairments for caregivers, particularly as it may relate to burden. It is important to note that these studies were not conducted in Latin America or with diverse samples. As such, future research should seek to determine if these interventions are also effective among individuals with PD and caregivers in other geographic regions and among racially/ethnically diverse samples.

The current study suggests that reducing caregiver burden is an important target for intervention as it channels directly into mental health. To date, there have been a number of interventions that target burden among caregivers of individuals with PD. One educational intervention that addressed the scheduling of pleasant activities, communication, reducing burden, and managing stress was shown to significantly reduce burden from baseline [56]. Further, interventions emphasizing education and fellowship with other caregivers have also been reported in the literature and have received qualitative support for reducing caregiver burden [18, 57, 58]. However, again, none of these interventions were conducted in Latin America and did not consist of diverse samples (in terms of race/ethnicity, languages spoken, etc.). Therefore, it is unclear if these interventions would be effective in this geographical region. As such, another critical target for intervention is the development and evaluation of interventions that may be culturally adapted for caregivers living in Latin America.

One such cultural consideration would be the influence of gender-role expectations as they contribute to caregiver burden. Links between female PD caregivers and increased anxiety have been found in some studies [20] while others have found no differences in mental health outcomes by sex [59]. However, there is evidence that Mexican gender-role expectations influence the amount and types (e.g., instrumental and emotional) of caregiving done by each sex; women take on markedly more caregiving duties than men, which may provide insight into potential lower mental health ratings [60]. Another cultural consideration is that the only demographic predictor of caregiver burden was education, such that higher education was associated with greater caregiver burden. This could be because caregivers with a higher educational level may have additional work-related duties that contribute unique stressors on top of heavy caregiving responsibilities. It would be important to consider whether educational attainment plays a differential role in caregiving in the U.S. versus Mexico, as U.S. caregivers have higher educational attainment. Future cross-cultural studies should attempt to tease out the contributions of gender roles and education in PD caregiver burden.

Given the demonstrated associations between mental health and deleterious outcomes for caregivers (e.g., burden), caregiver mental health may be an important intervention target. To date, at least one cognitive behavioral intervention has been shown to reduce caregiver burden among caregivers who report emotional distress. Secker and Brown [61] found that 12 to 14 sessions of cognitive behavioral therapy focused on relaxation, sleep hygiene, accessing support, and challenging negative beliefs delivered by a clinical psychologist reduced burden six months postintervention compared to the control group. Given the lack of mental health interventions for PD caregivers in Latin America, interventions that are culturally tailored for this population may serve to address the mental health needs of PD caregivers in a culturally sensitive manner.

### 4.1. Limitations and Future Research

#### 4.1.1. Methodological Weaknesses in Data Collection

The present study is limited in that it only recruited from two outpatient clinics: a specialty clinic in Henrico, Virginia, and a clinic in Guadalajara, Mexico. As such, the current study likely did not capture caregivers of individuals in the later stages of PD when individuals are likely to be institutionalized [62]. Given evidence demonstrating caregiver burden is highest in stage IV immediately prior to institutionalization at stage V [62], the relationships identified in the current study among outpatient caregivers may not generalize to all PD caregivers.

The data in the current study were collected using a slightly different methodology at the Mexico site and the United States site. At the United States site, caregivers completed the study measures independently using paper and pencil. In contrast, at the Mexico site, researchers used oral interviews to collect data from participants (in order to account for potential problems with illiteracy), which may have influenced the responses of participants. In addition, the study measures utilized in the current study (apart from the MDS-UPDRS) were validated for self-report and not for oral interviews. Therefore, it is possible that caregivers from the Mexico site responded differently from caregivers from the United States site.

Another limitation is that the data in this study were collected exclusively from caregivers. Therefore, the data in the current study represent their perceptions of PD-related impairments and caregiver burden and mental health. Future studies should also aim to use more objective measures such as patient medical records to assess PD-related impairments. It may be particularly helpful to collect objective information on disease stage, which has been associated with deleterious outcomes for caregivers such as burden [21] and which unfortunately was not collected in the current study. Furthermore, collecting data regarding whether or not the patient is currently taking PD medication may be informative, as dopamine agonists and L-dopa (which are often used to address PD symptomatology) may have a number of side effects. The unintended effects of these pharmaceutical agents may include nausea, impulsive behaviors (including impulse control disorders), dopamine dysregulation syndrome, and psychosis [63], which may result in negative consequences for the caregiver such as lower quality of life [19, 20].

#### 4.1.2. Cross-Sectional Methodology

Since the current study was cross-sectional in nature, causal inferences cannot be made. Future research should utilize cross-lagged panel designs or other longitudinal methods to infer whether the relations identified in the current study may be causal in nature. Further, it is possible that some of the relationships are reciprocal. For example, it is possible that mental health problems also influence levels of caregiver burden.

#### 4.1.3. Generalizability

Given that the samples in the current study came from two clinics, it is possible that the experiences of these caregivers may not generalize to the population of caregivers of individuals living with PD in the United States and Mexico. For example, caregivers at the United States site were recruited from a PD specialty clinic in a suburban area, suggesting that the individuals they care for are at least receiving some health care. Similarly, participants from the Mexico site were recruited from a large, urban hospital, also suggesting they have at least some access to health care. The experiences of caregivers without health care, such as those in rural areas, may differ greatly from caregivers who do have health care.

## 5. Conclusions

The current study examined associations among PD-related impairments, caregiver burden, and caregiver mental health among caregivers of individuals with PD residing in the United States and Mexico. Findings from the current study as well as prior literature highlight the importance of targeting critical caregiver outcomes such as caregiver burden and caregiver mental health.

## Figures and Tables

**Table 1:** Characteristics of PD caregivers and patients.

Demographic variable	Value	
	United States	Mexico
Age		
Caregiver (years, mean (SD))	68.73 (8.36)	53.66 (14.96)
Patient (years, mean (SD))	71.61 (8.13)	65.68 (10.78)
Sex (%)		
Caregiver		
Female	68.6%	76.4%
Male	31.4%	23.6%
Patient		
Female	35.2%	48.0%
Male	64.8%	52.0%
Months providing care (mean (SD))	46.78 (81.33)	52.38 (49.22)
Relationship to individual with PD (%)		
Parent	3.8%	34.5%
Aunt/uncle	1.0%	1.4%
Spouse/romantic partner	93.3%	51.4%
Sibling	0%	7.4%
Child	0%	0%
Friend	1.9%	.7%
Cousin	0%	.7%
Other	0%	4.1%

**Table 2:** Summary of symptoms reported by caregivers (*N* = 253).

Symptom domain	Symptom endorsed	% endorsing symptom	
		United States	Mexico
Nonmotor	Cognitive impairment		
	Normal	26.7%	39.2%
	Slight	27.6%	29.1%
	Mild	17.1%	15.5%
	Moderate	23.8%	11.5%
	Severe	4.8%	4.7%
	Hallucinations and psychosis		
	Normal	78.1%	82.4%
	Slight	12.4%	9.5%
	Mild	5.7%	4.1%
	Moderate	2.9%	2.7%
	Severe	1.0%	1.4%
	Depressed mood		
	Normal	37.1%	33.1%
	Slight	41.0%	24.3%
	Mild	11.4%	17.6%
	Moderate	8.6%	20.3%
	Severe	1.9%	4.7%
	Anxious mood		
	Normal	36.2%	31.3%
	Slight	38.1%	31.8%
	Mild	17.1%	16.2%
	Moderate	6.7%	18.2%
	Severe	1.9%	2.7%
	Apathy		
	Normal	39.0%	56.1%
	Slight	35.2%	17.6%
	Mild	18.1%	13.5%
	Moderate	3.8%	10.5%
	Severe	3.8%	2.7%
	Dopamine dysregulation syndrome		
	Normal	68.6%	79.1%
	Slight	16.2%	8.1%
	Mild	11.4%	5.4%
	Moderate	3.8%	4.7%
	Severe	0.0%	2.7%
	Sleep problems		
	Normal	19.0%	33.1%
	Slight	22.9%	20.9%
	Mild	27.6%	16.9%
	Moderate	23.8%	19.6%
	Severe	5.7%	9.5%
	Daytime sleepiness		
	Normal	14.3%	29.1%
	Slight	24.8%	25.0%
	Mild	51.5%	25.0%
	Moderate	6.7%	16.9%
	Severe	2.9%	4.1%
	Pain and other sensations		
	Normal	21.9%	30.4%
	Slight	36.2%	31.3%
	Mild	15.3%	21.6%
	Moderate	16.2%	14.2%
	Severe	10.5%	2.7%
	Urinary problems		
	Normal	38.1%	54.1%
	Slight	26.7%	23.0%
	Mild	15.2%	10.1%
	Moderate	13.3%	9.5%
	Severe	6.7%	3.4%
	Constipation		
	Normal	33.3%	43.2%
	Slight	37.1%	23.0%
	Mild	16.2%	18.9%
	Moderate	13.3%	12.8%
	Severe	0.0%	2.0%
	Lightheadedness on standing		
	Normal	52.4%	65.5%
	Slight	27.6%	19.6%
	Mild	10.5%	5.4%
	Moderate	9.5%	8.1%
	Severe	0.0%	1.4%
	Fatigue		
	Normal	18.1%	34.5%
	Slight	41.0%	27.7%
	Mild	25.7%	18.2%
	Moderate	10.5%	14.2%
	Severe	4.8%	5.4%

Motor	Speech		
	Normal	24.8%	38.5%
	Slight	19.0%	25.7%
	Mild	29.5%	16.9%
	Moderate	23.8%	12.8%
	Severe	2.9%	6.1%
	Saliva/drooling		
	Normal	57.1%	60.8%
	Slight	12.4%	13.5%
	Mild	11.4%	16.2%
	Moderate	9.5%	8.1%
	Severe	9.5%	1.4%
	Chewing and swallowing		
	Normal	55.2%	72.3%
	Slight	36.2%	10.8%
	Mild	6.7%	10.8%
	Moderate	1.0%	4.7%
	Severe	0.0%	1.4%
	Eating tasks		
	Normal	45.7%	43.2%
	Slight	28.6%	33.1%
	Mild	21.9%	16.2%
	Moderate	2.9%	6.8%
	Severe	1.0%	0.7%
	Dressing		
	Normal	28.6%	22.3%
	Slight	21.0%	52.0%
	Mild	37.1%	15.5%
	Moderate	7.6%	7.4%
	Severe	5.7%	2.7%
	Hygiene		
	Normal	42.9%	27.7%
	Slight	31.4%	49.3%
	Mild	17.1%	15.5%
	Moderate	4.8%	4.1%
	Severe	3.8%	3.4%
	Handwriting		
	Normal	22.9%	31.8%
	Slight	25.7%	33.8%
	Mild	22.9%	14.9%
	Moderate	21.0%	11.5%
	Severe	7.6%	8.1%
	Hobbies and other activities		
	Normal	27.6%	27.7%
	Slight	20.0%	21.6%
	Mild	25.7%	15.5%
	Moderate	17.1%	23.0%
	Severe	9.5%	12.2%
	Turning in bed		
	Normal	38.1%	37.2%
	Slight	41.9%	35.8%
	Mild	12.4%	16.2%
	Moderate	5.7%	7.4%
	Severe	1.9%	3.4%
	Tremor		
	Normal	26.7%	18.9%
	Slight	46.7%	44.6%
	Mild	18.1%	18.2%
	Moderate	6.7%	14.2%
	Severe	1.9%	4.1%
	Getting out of bed, car, or deep chair		
	Normal	14.3%	31.1%
	Slight	34.3%	28.4%
	Mild	29.5%	25.0%
	Moderate	15.2%	10.8%
	Severe	6.7%	4.7%
	Walking and balancing		
	Normal	14.3%	23.6%
	Slight	42.0%	39.9%
	Mild	14.3%	16.2%
	Moderate	24.8%	15.5%
	Severe	4.8%	4.7%
	Freezing		
	Normal	52.4%	60.8%
	Slight	20.0%	18.9%
	Mild	6.7%	6.8%
	Moderate	15.2%	8.8%
	Severe	5.7%	4.7%

## Data Availability

The data used to support the findings of this study are available from the corresponding author upon request.
